# Multidisciplinary Prophylactic Strategies for Recurrence of Laryngeal Edema After Tooth Extraction in a Patient With Hereditary Angioedema: A Case Report

**DOI:** 10.7759/cureus.46869

**Published:** 2023-10-11

**Authors:** Daisuke Honda, Isao Ohsawa, Masashi Aizawa, Isao Miyamoto, Katsuhiro Uzawa, Katsuhiko Asanuma

**Affiliations:** 1 Department of Nephrology, Graduate School of Medicine, Chiba University, Chiba, JPN; 2 Department of Internal Medicine, Nephrology Unit, Saiyū Sōka Hospital, Sōka, JPN; 3 Department of Oral-Maxillofacial Surgery, Chiba University Hospital, Chiba, JPN

**Keywords:** recurrence, acute attack, trigger, tooth extraction, laryngeal edema, prophylaxis, hereditary angioedema

## Abstract

Hereditary angioedema (HAE), caused by C1-inhibitor (C1-INH) deficiency or dysfunction, is a rare and potentially life-threatening disease that leads to unpredictable recurrent attacks of angioedema in localized regions, including the larynx. As medical or dental procedures can trigger laryngeal edema, resulting in asphyxiation, major global guidelines recommend short-term prophylaxis prior to invasive procedures and long-term prophylaxis to prevent acute attacks and achieve near-normal lives. Here, we report the case of a 63-year-old male who experienced asphyxiation after tooth extraction. Emergency tracheotomy had saved his life at the age of 40 years, before the diagnosis of HAE. At the age of 63, when he had another opportunity for tooth extraction, he was definitively diagnosed with HAE. Administering short-term prophylaxis with ongoing long-term prophylaxis for HAE and perioperative multidisciplinary management for tooth extraction helped prevent recurrent fatal angioedema due to dental procedures and this can be useful when managing patients with HAE.

## Introduction

Hereditary angioedema (HAE), which is primarily caused by C1-inhibitor (C1-INH) deficiency or dysfunction, is a rare autosomal dominant disease. C1-INH deficiency or dysfunction can lead to a paroxysmal increase in the production of bradykinin, a vasoactive peptide that causes acute and recurrent attacks of localized subcutaneous or submucosal angioedema in the extremities, face, gastrointestinal tract, and larynx [1,2]. Untreated upper airway edema may lead to fatal asphyxia [3]. Angioedema attacks can be triggered by stress, trauma, infection, drugs, surgeries, and dental procedures, and can last two to four days if left untreated [4].

The World Allergy Organization/European Academy of Allergy and Clinical Immunology (WAO/EAACI) guidelines recommend on-demand treatment for acute attacks and short- and long-term prophylaxis for suppressing the aggravation of symptoms and preventing the recurrence of an acute attack [5]. The goals of treatment are to achieve complete disease control and normalize the lives of patients. In Japan and Western countries, therapeutic approaches for acute attacks (on-demand treatment) and the prophylaxis of HAE have been developing rapidly. Such medications are available for patients diagnosed with HAE. Although HAE is estimated to affect approximately one in 50,000 individuals, fewer patients than the estimated prevalence have been diagnosed worldwide, mainly owing to low awareness of the disease, which is a major clinical issue despite the readily available therapeutic options [6].

Several studies have shown that patients with HAE experience asphyxiation due to laryngeal edema during the undiagnosed period [7-9]. For the clinical management of HAE, early diagnosis and prompt treatment during acute attacks are important to decrease mortality and morbidity [10]. Here, we report a case of asphyxiation due to laryngeal edema after tooth extraction. Emergency tracheotomy prevented death before the diagnosis of HAE. Before further tooth extraction, he was prescribed appropriate prophylaxis, and perioperative management contributed to the successful prevention of the recurrence of fatal angioedema due to dental procedures.

## Case presentation

We present the case of a 63-year-old male patient with HAE. The patient first became aware of edema in the extremities in junior high school and continued to have recurrent local edema throughout his body. At the age of 40 years, he experienced laryngeal edema and suffocated after tooth extraction (left maxillary third molar) but was saved by an emergency tracheotomy. Subsequently, the patient avoided invasive dental procedures due to fear of asphyxiation recurrence. He had a desk job and experienced two to three attacks per month, mainly on his hands. He knew that his father and older brother had been diagnosed with HAE, but he had not been definitively diagnosed.

**Figure 1:**
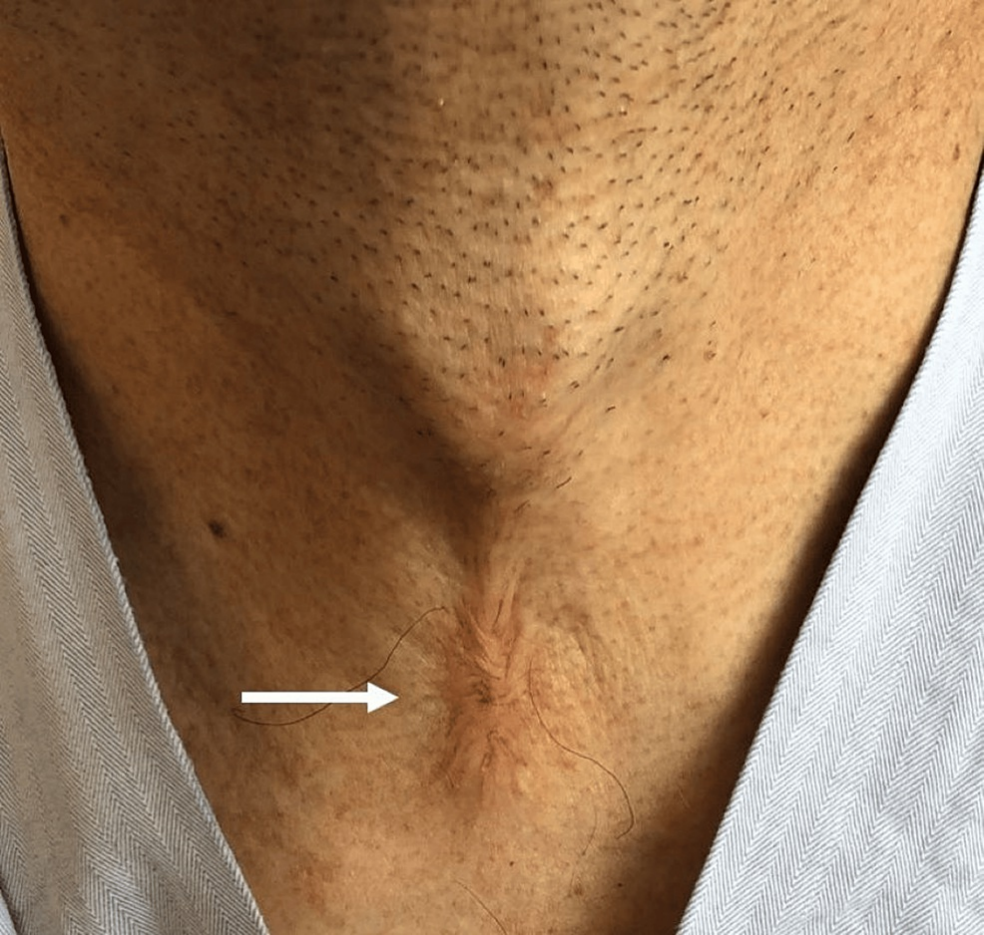
Visible scar on the anterior region of the neck of the patient (white arrow) of tracheotomy performed for asphyxiation due to laryngeal edema after tooth extraction 23 years prior

In September 2022, the patient was referred to the Department of Oral-Maxillofacial Surgery and the Department of Nephrology for dental treatment for fourth-degree dental caries in the right mandibular second molar and further examination of suspected HAE. Blood tests revealed C1-INH function of <25% (reference value: 70-130%) and C4 of 2 mg/dL (reference value: 17-45 mg/dL), and he was accurately diagnosed with HAE based on personal and family history, and the laboratory findings. During his visit, a scar due to the past tracheotomy was observed (Figure 1), and he had been taking linagliptin, metformin, and allopurinol for diabetes and hyperuricemia, as prescribed by his primary care physician.

Considering the high risk during extraction following the HAE diagnosis, we delayed the procedure. Furthermore, we requested the primary care physician to change linagliptin, which is a bradykinin-cleaving enzyme inhibitor that provokes and exacerbates angioedema attacks, to a suitable alternative [11]. The patient applied to the Medical Expense Subsidy System for Specific Diseases (designated intractable diseases) by the Japanese government to reduce the financial burden related to the cost of HAE treatment. After the application was approved in January 2023, he started using 150 mg of berotralstat (a kallikrein inhibitor) as a long-term prophylactic oral agent, which resulted in the disappearance of his acute HAE attacks. We also trained him to self-administer icatibant (a bradykinin B2 receptor antagonist) as an on-demand treatment and prescribed it to him.

The patient was admitted to our hospital one day before tooth extraction in April 2023 as a high-risk case. On the day of tooth extraction, he took 150 mg of berotralstat in the morning, and 1500 U of C1-INH concentrate (21.6 U/kg) was administered two hours before the invasive dental procedures as short-term prophylaxis. Intravenous tranquilizers (1 mg of midazolam and 20 mg of propofol) were administered in the operating room by anesthesiologists, and local anesthesia with 3% concentrated propitocaine was used. Dentists extracted the right mandibular second molar with fourth-degree dental caries without any problems. The patient was transferred to the high-dependency unit with an intravenous line and maintained for observation after the dental procedure. Consequently, the patient was discharged from the hospital the next day without recurrence of an acute attack of HAE.

## Discussion

HAE is a life-threatening disease caused by untreated acute attacks in the upper airways [12]. Patients with HAE are often forced to restrict their behavior not only during acute attacks but also during non-attack periods owing to anxiety about the recurrence of acute attacks [13]. Therefore, the WAO/EAACI guidelines describe the importance of long-term prophylaxis and on-demand treatment to achieve a near-normal life [5]. They also describe the utility and necessity of short-term prophylaxis prior to invasive medical or dental procedures. There are many reports on successful perioperative management of patients with HAE by short-term prophylaxis [12,14,15]. However, the WAO/EAACI guidelines include no detailed description of short-term prophylaxis under long-term prophylaxis and encourage studies and reports on the need for short-term prophylaxis in patients currently on long-term prophylaxis [5].

In the current case, the medical team in our hospital made various efforts with the ultimate common goal of preventing the recurrence of laryngeal edema due to tooth extraction because the patient experienced life-saving treatment owing to asphyxiation after the previous tooth extraction, which is reported to be one of the risk factors recurrently causing laryngeal edema [16]. First, HAE specialists appropriately made a diagnosis and provided total management for HAE. The patient received berotralstat (150 mg/day) as long-term prophylaxis and 1500 U of C1-INH concentrate (21.6 U/kg) two hours prior to the invasive dental procedure as short-term prophylaxis. C1-INH function was less than 25% at the first visit but increased to 68% in the blood samples at the beginning of the dental procedures. It has been reported that long-term prophylaxis by C1-INH concentrate significantly reduces the incidence of acute attacks when C1-INH function continuously exceeds 40% [17]. Thus, we can consider that short-term prophylaxis with C1-INH concentrate under long-term prophylaxis with berotralstat in this case could partially contribute to preventing the recurrence of laryngeal edema after tooth extraction. We were also ready to administer C1-INH concentrate and icatibant as on-demand treatment during his hospitalization. Further, dentists performed tooth extraction with minimum trauma to surrounding structures, anesthesiologists managed the intravenous anesthesia, and emergency physicians were prepared to perform life-saving tracheal intubation or tracheotomy procedures when laryngeal edema appeared. Thus, collaboration with the relevant hospital departments is important in situations in which fatal acute attacks may occur. Educating patients to report any abnormalities as soon as possible is advised, as is regular monitoring of objective indicators, such as vital signs. Although tooth extraction is performed in patients with undiagnosed HAE, which can trigger laryngeal edema, we believe that it is important for physicians and dentists to always recognize HAE and ask patients with a history of angioedema to rule out HAE before invasive medical and dental procedures (Table 1).

**Table 1:** Multidisciplinary prophylactic strategies for recurrence of laryngeal edema after tooth extraction HAE, hereditary angioedema; C1-INH, C1-inhibitor

Who	Aim	Method
Whole team	To prevent recurrence of a fatal acute attack	Each specialist developed individual strategy
HAE specialist	To provide total management for HAE treatment and to prescribe medicines	Made a definitive diagnosis of HAE
		Requested primary care physician to change linagliptin
		Helped patient apply to the Japanese government's medical expense subsidy system
		Trained him to self-administer icatibant as on-demand treatment in case of an acute attack
		Prescribed berotralstat as long-term prophylaxis
		Administered C1-INH concentrate before invasive dental procedures as short-term prophylaxis
		Prepared for C1-INH concentrate and icatibant as an on-demand treatment during his hospitalization
Dentist	To perform tooth extraction with the smallest invasion	Finished the procedure in a short time and enough local anesthesia with propitocaine
Anesthesiologist	To sedate during dental procedures	Managed the intravenous anesthesia with midazolam and propofol
Emergency physician	To prepare for prompt emergency response in case of laryngeal edema	Monitored his postoperative course in the high-dependency unit
Patient	To report any abnormalities in himself as soon as possible	Educated to monitor himself and report abnormalities

## Conclusions

HAE is a potentially fatal disease since upper airway edema may lead to asphyxia. Thus, the goals of HAE treatment are to achieve complete disease control and normalize the lives of patients. Necessary medical or dental procedures sometimes may trigger angioedema attacks of HAE. However, recurrent laryngeal edema due to dental procedures can be prevented with proper diagnosis, prophylaxis, and multidisciplinary perioperative management, even in patients with HAE who have previously suffered from laryngeal edema and asphyxia due to tooth extraction.

## References

[1] Longhurst H, Cicardi M (2012). Hereditary angio-oedema. Lancet.

[2] Bork K, Meng G, Staubach P, Hardt J (2006). Hereditary angioedema: new findings concerning symptoms, affected organs, and course. Am J Med.

[3] Honda D, Ohsawa I, Shimizu Y (2018). Suffocation due to acute airway edema in a patient with hereditary angioedema highlighted the need for urgent improvements in treatment availability in Japan. Intern Med.

[4] Zotter Z, Csuka D, Szabó E (2014). The influence of trigger factors on hereditary angioedema due to C1-inhibitor deficiency. Orphanet J Rare Dis.

[5] Maurer M, Magerl M, Betschel S (2022). The international WAO/EAACI guideline for the management of hereditary angioedema - the 2021 revision and update. World Allergy Organ J.

[6] Ohsawa I, Honda D, Nagamachi S (2015). Clinical manifestations, diagnosis, and treatment of hereditary angioedema: survey data from 94 physicians in Japan. Ann Allergy Asthma Immunol.

[7] Forrest A, Milne N, Soon A (2017). Hereditary angioedema: death after a dental extraction. Aust Dent J.

[8] Jacobs J (2011). Recurrent attacks of hereditary angioedema: a case of delayed diagnosis. Allergy Asthma Proc.

[9] Balla Z, Andrási N, Pólai Z (2021). The characteristics of upper airway edema in hereditary and acquired angioedema with C1-inhibitor deficiency. Clin Transl Allergy.

[10] Honda D, Ohsawa I, Iwanami K, Rinno H, Tomino Y, Suzuki Y (2021). A case of hereditary angioedema due to C1-inhibitor deficiency with recurrent abdominal pain diagnosed 40 years after the occurrence of the initial symptom. Clin J Gastroenterol.

[11] Byrd JB, Touzin K, Sile S (2008). Dipeptidyl peptidase IV in angiotensin-converting enzyme inhibitor associated angioedema. Hypertension.

[12] Sanuki T, Watanabe T, Kurata S, Ayuse T (2014). Perioperative management of tooth extractions for a patient with hereditary angioedema. J Oral Maxillofac Surg.

[13] Yamamoto B, Honda D, Ohsawa I (2023). Burden of illness seen in hereditary angioedema in Japanese patients: results from a patient reported outcome survey. Intractable Rare Dis Res.

[14] Tanaka KA, Mondal S, Morita Y, Williams B, Strauss ER, Cicardi M (2020). Perioperative management of patients with hereditary angioedema with special considerations for cardiopulmonary bypass. Anesth Analg.

[15] Adami EC, Magri F, Plotti C, Renzi S, Chiarini G, De Cicco G (2023). Hereditary angioedema in cardiac surgery: perioperative management considerations for a rare disease. Perfusion.

[16] Bork K, Hardt J, Schicketanz KH, Ressel N (2003). Clinical studies of sudden upper airway obstruction in patients with hereditary angioedema due to C1 esterase inhibitor deficiency. Arch Intern Med.

[17] Longhurst H, Cicardi M, Craig T (2017). Prevention of hereditary angioedema attacks with a subcutaneous C1 inhibitor. N Engl J Med.

